# Mesoscopic Energy Minimization Drives Pseudomonas aeruginosa Biofilm Morphologies and Consequent Stratification of Antibiotic Activity Based on Cell Metabolism

**DOI:** 10.1128/AAC.02544-17

**Published:** 2018-04-26

**Authors:** M. V. Sheraton, J. K. H. Yam, C. H. Tan, H. S. Oh, E. Mancini, L. Yang, S. A. Rice, P. M. A. Sloot

**Affiliations:** a Complexity Institute, Nanyang Technological University, Singapore; b HEALTHTECH NTU, Interdisciplinary Graduate School, Nanyang Technological University, Singapore; c Singapore Centre for Environmental Life Sciences Engineering, Nanyang Technological University, Singapore; d School of Materials Science and Engineering, Nanyang Technological University, Singapore; e Institute for Advanced Study, University of Amsterdam, Amsterdam, The Netherlands; f The School of Biological Sciences, Nanyang Technological University, Singapore; g The ithree Institute, The University of Technology, Sydney, NSW, Australia; h ITMO University, Saint Petersburg, Russian Federation

**Keywords:** mushroom-shaped biofilm, cellular Potts model, chemotaxis, Pseudomonas aeruginosa, antibiotic resistance, biofilms, cell motility, cell proliferation

## Abstract

Segregation of bacteria based on their metabolic activities in biofilms plays an important role in the development of antibiotic resistance. Mushroom-shaped biofilm structures, which are reported for many bacteria, exhibit topographically varying levels of multiple drug resistance from the cap of the mushroom to its stalk. Understanding the dynamics behind the formation of such structures can aid in design of drug delivery systems, antibiotics, or physical systems for removal of biofilms. We explored the development of metabolically heterogeneous Pseudomonas aeruginosa biofilms using numerical models and laboratory knockout experiments on wild-type and chemotaxis-deficient mutants. We show that chemotactic processes dominate the transformation of slender and hemispherical structures into mushroom structures with a signature cap. Cellular Potts model simulation and experimental data provide evidence that accelerated movement of bacteria along the periphery of the biofilm, due to nutrient cues, results in the formation of mushroom structures and bacterial segregation. Multidrug resistance of bacteria is one of the most threatening dangers to public health. Understanding the mechanisms of the development of mushroom-shaped biofilms helps to identify the multidrug-resistant regions. We decoded the dynamics of the structural evolution of bacterial biofilms and the physics behind the formation of biofilm structures as well as the biological triggers that produce them. Combining *in vitro* gene knockout experiments with *in silico* models showed that chemotactic motility is one of the main driving forces for the formation of stalks and caps. Our results provide physicists and biologists with a new perspective on biofilm removal and eradication strategies.

## INTRODUCTION

Bacteria thrive in natural environments using two modes of growth, (i) planktonic growth by independent, single bacteria and (ii) biofilm growth, in which the cells function as a group. Planktonic bacteria proliferate, infect hosts, and move without much physical interaction with other bacteria in their vicinity. They are vulnerable to antibiotics and to bacteriophages in their vicinity. In contrast, bacteria have evolved the ability to aggregate together as biofilms to protect themselves from predators and reduce the threats from antibiotics or toxic substances. Once a biofilm is established, it can host billions of bacteria that function communally. However, bacterial cells within a single biofilm exhibit different physiological states. They can be alive and active, alive and metabolically less active (dormant), or dead and decaying in different parts inside the biofilm (1, 2). Some bacteria in biofilms are known to develop resistance to multiple antibiotics (3, 4). For example, cells present at the top or cap of mushroom-shaped biofilms have been shown to be resistant to colistin (5). Cells within or on the stalk of mushroom-shaped biofilms, however, have shown resistance to carbapenems and tobramycin (6). This suggests that it is impossible to eliminate the entire mushroom structure using a single drug. Even worse, such efforts could lead to selective killing of non-drug-resistant bacteria, leaving behind the drug-resistant strains and accelerating the spread of an infection. In a few cases, it has been shown that dormant bacteria are resistant to antibiotic treatments; therefore, the segregation of bacteria into different states within the biofilm will lead to differential drug resistance expression at different regions (7).

Recently, the World Health Organization (8) published a list of 12 bacteria which could be of great threat to human health due to their multidrug resistance. Pseudomonas aeruginosa has been identified as one of the bacteria of critical priority. P. aeruginosa is known to form mushroom-shaped biofilm structures in nature and during spaceflights (9). Cells in the interior of the mushroom-shape biofilm have low metabolic activity, while the cells near the cap of the mushroom-shape biofilm have high metabolic activity (10, 11). They can exhibit multidrug resistance within the same mushroom structure as a consequence of these microcolonies harboring cells in different metabolic states (12). Thus, due to the differences in physiological status among the cells within the biofilm, it is difficult to eradicate the biofilm via drug monotherapy. For example, colistin selectively kills less active cells (11, 13, 14), while tobramycin kills highly active cells in the biofilm (15, 16). While some studies have focused on microcolony formation, the mechanisms and dynamics of microcolony formation are currently not well understood (17–20). If a mixture of two strains of P. aeruginosa bacteria, e.g., a wild type with motility and a nonmotile mutant, are cultured together, mushroom structures are formed with the wild-type motile bacteria on the cap of the mushroom and the nonmotile mutants occupying the stalk of the mushroom (19). Modeling studies have shown that bacterial motility plays a major role in determining the shape of the biofilm structure. Farrell et al. (21), developed a quasi-two-dimensional force-based biofilm growth model to study the branching of biofilms consisting of nonmotile bacteria. It was shown numerically that mechanical interactions between the bacteria lead to the formation of two-dimensional (2D) finger-like biofilm structures, which was previously thought to be an outcome of diffusion limitation. This observation suggests that the macroscopic biofilm structure is actively changed by microscopic interactions between individual bacteria and is not a passively evolved structure due to nutrient gradients. However, extensive studies considering interaction forces between the bacteria and nutrient limitations were unable to predict the formation of the observed complex 3D mushroom shapes (21–25). Typically, these studies predicted a series of hemispherical shapes but were not able to predict the mushroom shapes observed in nature, specifically with those involving wild-type bacteria. Here we report on the dynamics of biofilm shapes as they are influenced by the availability of nutrients, the distribution of motile cells, and cell-cell interactions through volume and chemotactic forces. Using laboratory experiments coupled with *in silico* numerical studies, we identified the key parameters that determine the thickness and height of the stalk as well as the cap of these macroscopic structures and consequently the distribution of dormant and metabolically active bacteria. The laboratory experiments, utilizing wild-type bacteria and specific mutants, were used to quantify and validate the outcomes of the biofilm growth simulation model. We also show that chemotactic processes dominate the transformation of slender and hemispherical structures to mushroom structures with a signature cap.

### Simulation model.

Each bacterium in the simulation is considered a collection of pixels. As the mass increases, the number of pixels for each cell increases proportionally and the cells divide once the number of pixels has doubled. The cells' mass increment is modeled using Tessier kinetics (26). We consider two nutrients, glucose and oxygen, diffusing from the top of the simulation domain. Glucose is present in excess in both the experiments and the numerical models. Single-solute Monod kinetics is the common choice in most biofilm models (25, 27–29). This, however, would result in exponential cell growth in the simulations due to the presence of excess nutrients. Double-solute Tessier kinetics models the bacterial mass increase in a more realistic way than single-solute Monod kinetics by establishing a nutrient consumption rate that is dependent on both limiting and excess nutrients, thereby preventing exponential cell proliferation. It has been shown in previous studies that Tessier kinetics models the growth of P. aeruginosa biofilms more accurately than Contois, Monod, or other combined kinetics (26, 30). We have therefore developed two-substrate Tessier kinetics for modeling the biofilm growth from uptake of oxygen and glucose (Table 1). Our model based on Tessier kinetics is a more accurate predictor of the proliferation rate of bacterial cells than are previous P. aeruginosa biofilm simulations (25, 27–29); the proliferation rate is an important parameter for transformation of cells from an active state to a dormant state.

(1) $$\frac{\partial S_{o}}{\partial t}=D_{o}\left(\frac{\partial^{2}S_{o}}{\partial x^{2}}+\frac{\partial^{2}S_{o}}{\partial y^{2}}+\frac{\partial^{2}S_{o}}{\partial z^{2}}\right)-r_{o}(S_{o},S_{g},B_{C})$$

(2) $$\frac{\partial S_{g}}{\partial t}=D_{g}\left(\frac{\partial^{2}S_{g}}{\partial x^{2}}+\frac{\partial^{2}S_{g}}{\partial y^{2}}+\frac{\partial^{2}S_{g}}{\partial z^{2}}\right)-r_{g}(S_{o},S_{g},B_{C})$$

(3) $$r_{o}\left(S_{o},S_{g},B_{C}\right)=\left[\frac{\mu \left(1-e^{\frac{C_{o}}{K_{o}}})(1-e^{\frac{C_{g}}{K_{g}}}\right)}{Y_{o}}+m_{o}\right]B_{c}$$

(4) $$r_{g}\left(S_{o},S_{g},B_{C}\right)=\left[\frac{\mu \left(1-e^{\frac{C_{o}}{K_{o}}}\right)\left(1-e^{\frac{C_{g}}{K_{g}}}\right)}{Y_{g}}+m_{g}\right]B_{c}$$

**TABLE 1:** Summary of the values of different parameters used in the Tessier kinetics model simulations

Parameter	Value^*a*^
Domain size	150 × 150 × 150 μm
Initial mass of bacteria (27)	1.315 × 10^−13^ g
Initial vol of bacteria (27)	27 μm^3^
No. of initial bacteria	5 cells
Half-saturation coefficient of glucose (26)	26.9 g m^−3^
Half-saturation coefficient of oxygen (26)	1.18 g m^−3^
Boundary layer thickness (27)	16.5 μm
Diffusion coefficient of glucose	2.52 × 10^−6^ m^2^ h^−1^
Diffusion coefficient of oxygen	7.2 × 10^−6^ m^2^ h^−1^
Maintenance coefficient for glucose (26)	0.0078 g g_b_^−1^ h^−1^
Maintenance coefficient for oxygen (26)	0.014 g g_b_^−1^ h^−1^
Specific growth rate (27)	0.29 h^−1^
Yield coefficient of oxygen (26)	0.635
Yield coefficient of glucose (26)	0.628
Chemotaxis potential	400
Fluctuation amplitude term	40
Initial glucose concn	400 g m^−3^
Initial oxygen concn	8 g m^−3^

a g_b_, gram biomass.

Equations 1 and 2 describe the time evolution of nutrient concentrations in the simulation domain, and equations 3 and 4 quantify the rate of nutrient consumption by the biomass. The equations are solved until steady state is reached, and the concentration at steady state is used to estimate the mass increase through equation 7. The motile bacteria can move through the domain between each time step of nutrient estimation, which is 1 h. The motility of the cells is based on their energy constraint, based on the Glazier-Graner-Hogeweg (GGH) model (31, 32) given by equation 8. Motility is then dependent on the volume constraints, chemotaxis, and contact adhesion between cells, substratum, and media. In the absence of volume increase and chemotaxis, this motility corresponds to bacterial random walks as observed in nature and is referred to as bacterial diffusion. The fluctuation amplitude term *T_m_* (membrane temperature) determines the average velocity of a random walk in the simulation. The value of *T_m_* is fixed in such a way that in the simulation the average distance moved by a cell in 1 h due to bacterial diffusion falls within the average distance covered by P. aeruginosa bacteria on a glass slide for 1 h in the experiments, which is around 145 μm/h (33).

*S_o_* and *S_g_* are the concentrations of oxygen and glucose, respectively, *D_o_* and *D_g_* are the diffusion coefficients of oxygen and glucose, respectively, μ is the cell growth rate, and *B_c_* is the biomass. The constants *K*, *Y*, and *m* are half-saturation, yield, and metabolic coefficients, respectively, with their subscripts indicating the corresponding substrate, oxygen or glucose. The mass increase as estimated by equation 7 is translated into a corresponding target volume (*V_T_*) increase, calculated from the mass density of the cells. The rate of volume increase or pixel addition to a cell is controlled by the change in energy shown in equation 9. In a sparsely populated space, a bacterium will be able to increase its volume faster than a bacterium in a densely packed space based on the volume constraint constant. Bacteria in tightly packed configurations, however, must push others toward the edge to increase their volume. Thus, the local energy interactions for pixel space allocation will result in an overall change in the structure of the biofilm. This energy interaction also prevents a cell from growing when there is no space to place the additional biomass, thus avoiding unrealistic cell proliferation. Therefore, the increase in biofilm biomass is controlled by nutrient consumption kinetics and by structural energy constraints within the biofilm.

(5) $$\frac{\partial B_{co}}{\partial t}=Y_{o}\left[r_{o}\left(S_{o},S_{g},B_{C}\right)-m_{o}B_{C}\right]$$

(6) $$\frac{\partial B_{cg}}{\partial t}=Y_{g}\left[r_{g}\left(S_{o},S_{g},B_{C}\right)-m_{g}B_{C}\right]$$

(7) $$\frac{\partial B_{c}}{\partial t}=\{\begin{array}{cc} \frac{\partial B_{co}}{\partial t}, & \frac{\partial B_{co}}{\partial t}<\frac{\partial B_{cg}}{\partial t}\\ \frac{\partial B_{cg}}{\partial t}, & \frac{\partial B_{co}}{\partial t}\geq \frac{\partial B_{cg}}{\partial t} \end{array}$$

*B_co_* and *B_cg_* are the biomass contributions from oxygen and glucose uptake, respectively.

(8) $$P\;\left[\sigma (\overset{\to }{\iota })\to \sigma (\overset{\to }{\iota^{\prime }})\right]=\{\begin{array}{cc} \left[exp\left(-\frac{\Delta H}{T_{m}}\right)\right], & \Delta H>0\\ 1\text{,} & \Delta H\leq 0 \end{array}$$

(9) $$\Delta E_{v}=\lambda {\left(V_{\text{cell}}-V_{T}\right)}^{2}$$

(10) $$\Delta E_{c}=\underset{i,j}{\sum }Con\left(\tau_{\sigma (i)},\tau_{\sigma (j)}\right)\;(1-\delta_{\sigma (i)}{{}_{,}}_{\sigma (j)})$$

The probability of a pixel copy (34) is calculated in equation 8. σ(*i*) is the pixel occupied by the cell, *V*_cell_ is the volume of the cell, and λ is the volume potential. Equation 10 describes the contact adhesion energy between the cells of different types τ, at positions *i* and *j*, where δ is the Kronecker delta function and *Con*(τ_σ(*i*)_, τ_σ(*j*)_) is the contact adhesion parameter. In the simulation, it is assumed that the cells are more adherent to the substratum than to each other, meaning less local energy change for adhering to the surface. The energy changes due to adhesion Δ*E_C_* and volume change Δ*E_V_* are combined to evaluate the total energy change, Δ*H* = Δ*E_V_ +* Δ*E_C_*.

## RESULTS AND DISCUSSION

**FIG 1:**
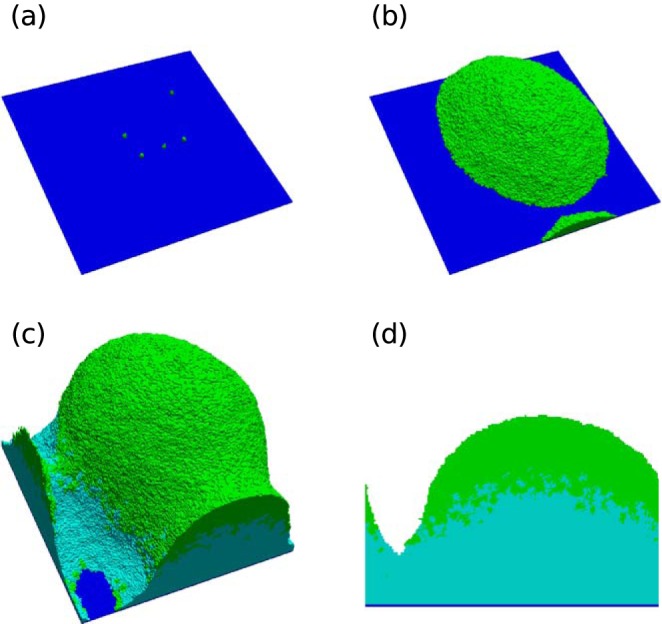
Simulated biofilm growth without chemotaxis. Shown are 3D views of the progress of biofilm development at 10 h (a), 30 h (b), and 50 h (c) and a 2D x-z cross section at 50 h (d). Green indicates active cells, and light blue indicates dormant cells.

**FIG 2:**
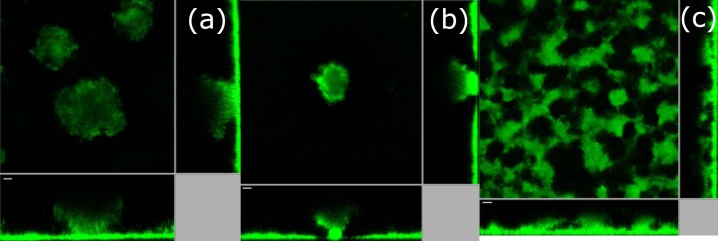
Confocal images for different strains of P. aeruginosa biofilms after 3 days. (a) Wild-type PAO1; (b) Δ*bdlA* dispersion mutant; (c) Δ*cheY* chemotaxis mutant. The scale bars are 20 μm.

The simulations were carried out for 50 simulation hours. The change in structure of the biofilm with time is summarized in Fig. 1. During the initial 20 h, the biofilm spreads itself across the surface because of the minimal energy change through bacterium-substratum contact. Later, when the nutrient concentration availability falls below the metabolic requirement of bacteria, the bacteria become dormant, indicated by the light blue color in the simulation. Dormant bacteria in their natural habitat generally are confined to their space without movement. In a similar way, dormant bacteria in the simulation lose their motility and their consumption rate becomes negligible. These processes add an extra layer to the growth segregation zone, the bottommost, no-growth dormant zone. Therefore, as time progresses the zones vary in thickness and, as expected, the final shape of the biofilm after 50 h was hemispherical. The preservation of segregation zones during the entirety of the growth process is due to the moving diffusion boundary layer and the increasing nutrient consumption rate at the dense lower layers. Most models in the literature, including the forced-based model (21), predict finger projection formation in 2D and simulate a hemisphere shape as shown in Fig. 1, rather than the mushroom shape observed in laboratory experiments with wild-type P. aeruginosa PAO1 bio1films (Fig. 2a). This indicates that some key mechanisms are missing and the current model is not capable of simulating the dynamics of mushroom shape formation. We performed experiments with a P. aeruginosa Δ*bdlA* dispersion mutant. We characterized the structures with clearly distinguishable stalks and caps, at least 70 μm tall, as mushroom-shaped biofilms. Δ*bdlA* mutants produce mushroom shapes as shown in Fig. 2b, suggesting that the ability of bacteria to leave the biofilm through active dispersal does not influence the formation of these mushroom shapes and that the formation is inherent in all wild-type P. aeruginosa bacteria irrespective of favorable or detrimental environmental cues.

As shown in Fig. 2, *bdlA* is not required for mushroom formation. Therefore, we carried out experiments using a Δ*cheY* chemotaxis mutant. These mutant lacks chemotactic motility, the motility associated with the directional movement of bacteria toward a nutrient presence. The biofilms produced by this Δ*cheY* mutant did not produce mushroom structures and instead formed large stalks without caps (Fig. 2c). This indicates a link between mushroom structure and chemotactic motility. The simulation model was modified accordingly to include bacterial chemotaxis. This chemotactic parameter was subsequently included as an energy term, Δ*E*, coupled with the already existing contact adhesion and volume constraint energy as Δ*H* = Δ*E_V_* + Δ*E_C_* + Δ*E*. The chemotactic energy potential satisfies three conditions, as follows.

If the critical chemotaxis concentration is zero, then the change in energy potential should be zero.

(11) $$C_{\text{sat}}\to 0,\Delta E\to 0$$

This condition biologically corresponds to the solute, which does not evoke chemotaxis in cells, ergo the critical chemotaxis concentration is zero.

For solute concentrations below the critical concentration *C_sat_*, the change in energy potential should be negative and chemotaxis should depend on the magnitude of the gradient in concentration.

(12) $$C_{\text{sat}}>S_{o}\left(\overset{\to }{i^{\prime }}\right),\Delta E<0$$

If the critical concentration is very high, then the change in energy potential should be minimum, which sets the cell to be always in motion along the gradient field with the minimum chemotaxis potential, 
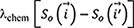
.

(13) $$C_{\text{sat}}\to \infty ,\Delta E\to \min$$

(14) $$\Delta E=\lambda_{\text{chem}}\left[\frac{S_{o}\left(\overset{\to }{i}\right)}{1+\frac{S_{o}\left(\overset{\to }{i}\right)}{C_{\text{sat}}}}-\frac{S_{o}\left(\overset{\to }{i^{\prime }}\right)}{1+\frac{S_{o}\left(\overset{\to }{i^{\prime }}\right)}{C_{\text{sat}}}}\right]$$

Equation 14 satisfies all three chemotaxis conditions and is used in the model to implement chemotaxis based on oxygen concentration. As mentioned above, an increase in volume of a cell in the simulation is modeled by increase in the number of pixels associated with the cell. The newly added pixels of a growing cell are placed in such a way that the local change in energy is minimized. Placing the new pixels along the oxygen gradient decreases the chemotactic energy and consequently the local energy change, thus favoring the cell growth along the nutrient gradient. It is expected that the bacteria in the biofilm will grow or move toward the nutrient enriched zone to sustain activity and biofilm growth. In the simulations, the motility is now a function of contact adhesion, volume constraint, and chemotaxis. Simulations with this new model with modified chemotaxis constraint produce mushroom shapes (Fig. 2). The potential, λ_chem_, determines the “chemotaxis velocity,” the velocity at which the bacteria move along the nutrient gradient. Higher chemotaxis velocity therefore thins out the stalk of the mushroom.

**FIG 3:**
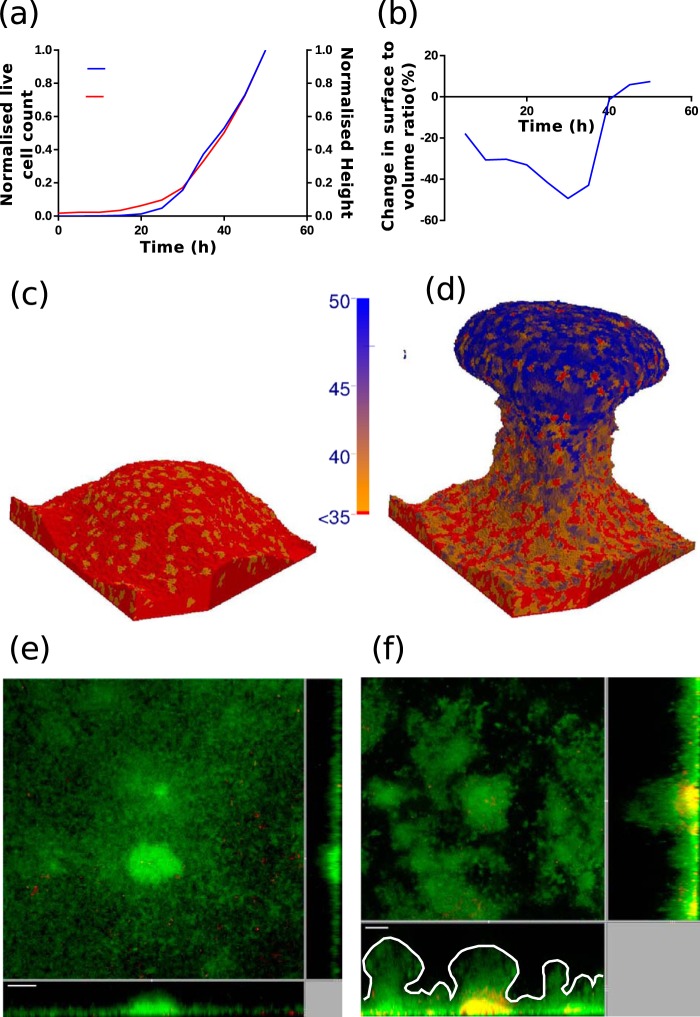
(a) Change of biofilm height and cell count with time. (b) Change in surface-to-volume ratio of the biofilm with time. (c and d) Simulation results showing the creation time (time at which a particular cell appears in the simulation for the first time) of the bacterial cells at different layers within the biofilm; the color key indicates the time of cell creation: 35 h (c) and 50 h (d). (e and f) Formation of mushroom structure of PAO1 wild-type biofilm after 1 day (e) and 3 days (f). Green color indicates live cells, red color indicates dead cells, and the white curve is a trace line on the outer surface of the biofilm.

**FIG 4:**
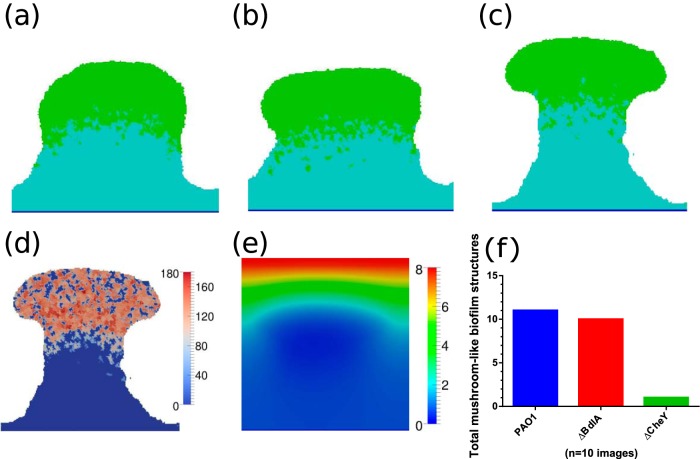
Simulated biofilm growth with modified chemotaxis. (a to c) Shown are 2D section views of the progress of biofilm development after 50 h at different λ_chem_ values: 0.75 λ_chem,fix_ (a), 1.0 λ_chem,fix_ (b) and 1.25 λ_chem,fix_ (c). Green indicates active cells, and light blue indicates dormant cells. (d to f) Shown are the distribution of cell motility in the model simulation with λ_chem_ being equal to 1.25 λ_chem,fix_ (d), distribution of oxygen concentration (e), and estimates of mushroom-shaped biofilm structures produced by different strains used in the experiments (f).

The change of the height of the biofilm and the number of live cells with time is shown in Fig. 3a. During the early stages of biofilm growth (20 to 30 h), the rate of height increase is significantly lower than the proliferation rate. This is due to the biofilm spreading across the substratum and covering a larger area, as was also observed in experimental data for day 1 biofilm (Fig. 3d). After 35 h, the height of the biofilm increases at a higher rate to accommodate the increase in total biomass. At the later stages of biofilm growth, even though the proliferation rate decreases, the height of biofilm increases exponentially. The critical point in time (35 h) after which the height increases exponentially is when the stalk of the mushroom starts to grow rapidly and a clear distinction between the cap and stalk appears. This critical point can be better estimated using the change in surface-to-volume ratio shown in Fig. 3b. After 35 h, the critical point, the surface area of the biofilm increases rapidly, leading to the formation of a broad cap at the top of mushroom. The spatial distribution of oxygen and the motility are shown in Fig. 4d and e, respectively. The cells at the bottom, which proliferate, must find a new space which is energetically favorable. The energetically favorable outcome for the cell is to move along the increasing nutrient gradient. In this GGH model with cell-cell adhesion energy, a cell in a crowded environment needs to expend more energy to push the nearby cells to move in its intended direction than does a cell in a sparsely populated environment. Due to this inherent density-controlled cell motility, the model favors the mobility of cells in the periphery of the biofilm.

Movement along the periphery of the microcolony is motion along the path of least resistance to minimize the local energy and, consequently, the global energy. As the cells in the periphery start to move upwards, the central width of the biofilm begins to thin, and the stalk starts to form in the middle (Fig. 3d). The mature cells, colored red in Fig. 3d, are found to climb over the relatively new cells at the stalk and periphery guided by the chemotaxis gradient. Once the chemotaxis potential has been maximized the motile cells start to aggregate into a crown at the top of the biofilm; in other words, the cells have reached the region where oxygen is available for survival. This process continues, and a clear distinction appears between the cap and the stalk part of the mushroom shape.

The model simulations without chemotaxis (Fig. 1) and the experiments using the chemotaxis-deficient Δ*cheY* mutant (Fig. 2c) did not produce any significant number of mushroom shaped structures, as shown in Fig. 4f. This clearly shows that chemotaxis is one of the key mechanisms in determining the shape of the biofilm. The formed structures in Δ*cheY* biofilms closely resemble the hemispherical shape formed by the contact and volume constraint version of the model. Segregation of bacteria within the biofilm based on their metabolic activity is conclusive from the model. In the simulations, three unique zones are observable in the formed mushroom structure: (i) the dormant bottom layers, (ii) nutrient-limited layers of the stalk, and (iii) fast-proliferating cells at the cap of mushroom. The bacteria in these three unique zones show differential responses to antibiotics. Consequently, the entire biofilm becomes highly heterogeneous over time, similar to the observations made by Williamson et al. (35). Therefore, eradication of the biofilm through clinical or chemical treatments is not straightforward due to the various levels of antibiotic resistance at different layers of the biofilm. This antibiotic resistance could arise due to the physiological heterogeneity (35) of the cells or the accumulation of genetic mutations based on the local stresses acting on the cells (36). Removing the cap and stalk of the mushroom will expose the dormant cells to a fresh nutrient supply. This would help them revert to a metabolically active state. In diseases, such as cystic fibrosis, involving P. aeruginosa biofilms, the reversion of dormant bacteria to an active state could result in exacerbations leading to acute infections (37). Our model can help us understand the time evolution of biofilm structure in P. aeruginosa biofilms and, along with it, the spatial distribution of antibiotic drug resistance. The model can be used to estimate antibiotic penetration and oxygen limitations, which have been shown (38) to be contributors of antibiotic tolerance in P. aeruginosa biofilms. Using model simulations to estimate the parameters, which could otherwise be hard or impossible to measure experimentally, will aid a clinician in understanding the inherent heterogeneity and provide valuable decision-making insights into selection of antibiotics. Additional development of the current model for other bacteria and inclusion of drug-induced cell lysis mechanisms can establish the model as a predictor of clinical efficacy of antibiotics.

## MATERIALS AND METHODS

### Bacterial strains and growth conditions.

The bacterial strains used in this study are listed in Table 2. P. aeruginosa strains were grown at 37°C in ABT minimal medium supplemented with 5 g liter^−1^ of glucose (ABTG) (39). Gentamicin (30 μg ml^−1^) or tetracycline (50 μg ml^−1^) was used as appropriate for marker selection in P. aeruginosa.

**TABLE 2:** Bacterial strains used in the experiments

Strains	Characteristic(s)^*a*^	Reference
PAO1	Wild type	41
Δ*bdlA*	PW3587 *bdlA*-F03::IS*lacZ*/hah *bdlA*-deficient strain; Tc^r^	42
Δ*cheY*	*cheY*-deficient strain; Gm^r^	43

a Tc^r^, tetracycline resistant; Gm^r^, gentamicin resistant.

### Cultivation of biofilms in flow chambers.

P. aeruginosa biofilms were cultivated in ABTG medium at 37°C using 40-mm by 4-mm by 1-mm three-channel flow chambers as previously described (40). Briefly, the bacterial strains were grown overnight in 2 ml of LB medium at 37°C with shaking (200 rpm). The overnight cultures were diluted 1:100 with ABTG medium, and 300 μl of the diluted culture was injected via syringe and needle into each channel. The ABTG medium flow was halted for 1 h for incubation of bacteria before resuming the flow at the rate of 4 ml h^−1^ using a Cole-Parmer Masterflex peristaltic pump (Cole-Parmer, United States) for development of biofilm. At each time point, 3.34 μM SYTO9 and 20 μM propidium iodide (PI) stains (LIVE/DEAD BacLight bacterial viability kit; Invitrogen) were injected into each channel to stain the biofilm for live and dead cell populations, respectively, for 15 min prior to confocal microscopy imaging. Experiments were performed in triplicate, and the representative images are shown as results.

### Confocal microscopy imaging.

The stained biofilm was observed under confocal laser scanning microscopy (CLSM) (LSM 780; Carl Zeiss, Germany), and images were acquired using either a ×20 or ×40 magnification objective lens. An argon laser (488 nm) and HeNe laser (561 nm) were used to observe the green and red fluorescence, respectively. The captured images were further processed using IMARIS software (Bitplane AG, Zurich, Switzerland) to generate the orthogonal view of the biofilm. Experiments were performed in triplicate, and the representative images are shown as results.
